# Long non‑coding RNA SNHG5 promotes osteogenic differentiation of human periodontal ligament stem cells via mediating miR‑23b‑3p/Runx2 axis

**DOI:** 10.7150/ijms.82454

**Published:** 2023-05-21

**Authors:** Xuefei Sun, Zhidan Li, Shaojie Dong, Qianqian Dong

**Affiliations:** 1 Key Laboratory of Shaanxi Province for Craniofacial Precision Medicine Research, College of Stomatology, Xi'an Jiaotong University, Xi'an, China.; 2 Clinical Research Center of Shaanxi Province for Dental and Maxillofacial Diseases, Department of Endodontics, College of Stomatology, Xi'an Jiaotong University, Xi'an, China.

**Keywords:** SNHG5, osteogenic differentiation, hPDLSCs (human periodontal ligament stem cells), miR‑23b‑3p

## Abstract

The treatment of bone loss due to periodontitis has posed a great challenge for physicians for decades. Therefore, it is of extraordinary significance to identify an effective regeneration scheme for alveolar bone. This study aimed to investigate long non-coding RNA (lncRNA) small nucleolar RNA host gene 5 (SNHG5) whether sponges microRNA-23b-3p (miR-23b-3p) to achieve the osteogenic differentiation of human periodontal ligament stem cells (hPDLSCs). Results revealed that the expression of SNHG5 was upregulated whereas that of miR-23b-3p was downregulated in osteogenic hPDLSCs. Alizarin red staining assays and qRT-PCR demonstrated that SNHG5 silencing or miR-23b-3p overexpression inhibits hPDLSCs osteogenic differentiation and vice versa. In addition, miR-23b-3p partially abolished the promotive effect of SNHG5 on osteogenic differentiation of hPDLSCs. Dual luciferase report and RNA pulldown assay verified that miR-23b-3p is a regulatory target of SNHG5 and that *Runx2* is a gene target of miR-23b-3p. In brief, the results demonstrate that SNHG5 promotes the osteogenic differentiation of hPDLSCs by regulating the miR-23b-3p/Runx2 axis. Our study provides novel mechanistic insights into the critical role of lncRNA SNHG5 as a miR-23b-3p sponge to regulate Runx2 expression in hPDLSCs and may serve as a potential therapeutics target for periodontitis.

## Introduction

Periodontitis is the most prevailing oral disease, which produces an enormous financial burden on public health 1. Severe periodontitis causes alveolar bone atrophy and absorption, which may lead to tooth loosening and loss. Therefore, on the basis of preventing lesions, alveolar bone regeneration is the key to the treatment of periodontitis. Recent studies have shown that long non-coding RNAs (lncRNAs) play a variety of biological functions to regulate gene expression through transcriptional regulation, post-transcriptional regulation and epigenetic regulation, and thus exert crucial roles in a variety of pathological processes, such as inflammation and bone metabolism 2-4. For example, lncRNA SNHG5 regulates the inflammatory response in status epilepticus rats through the nuclear factor-κB (NF-κB) signaling pathway 5. Feng et al. demonstrated that LncRNA XIST is involved in osteogenic differentiation of hPDLSCs through sponging miR-214-3p^6^. In addition, LncRNA SNHG5 promotes osteogenic differentiation of bone marrow mesenchymal stem cells through the miR-212-3p/GDF5/SMAD signaling axis 7. However, the role and mechanisms of SNHG5 during the osteogenic differentiation of hPDLSCs remain unclear.

Recent studies have demonstrated that LncRNAs are involved in regulating various biological processes through sponge miRNAs 8-10. The abnormal expression of miRNAs has also been reported to play an important role in the development of chronic periodontitis 11. Xu et al. reported that silencing of miR-132 increases osteoblastic differentiation of PDLSCs via activating NF-κB axis and targeting GDF5^12^. Another study found that lncRNA-ANCR directly targets miR-758 to regulate Notch2-Wnt/β-catenin signaling pathway and participate in PDLSCs bone formation 13. These recent findings suggest that miRNAs are involved in regulating the osteogenic differentiation of PDLSCs. Our recent studies have revealed that depletion of miR-23b reversed TNF-α-inhibited osteogenic differentiation of hPDLSCs by targeting Runx2^14^. It remains unclear whether SNHG5 regulates the osteogenic differentiation of PDLSCs by interacting with miR-23b.

In the present study, we attempted to expose the effect of SNHG5 on osteoblastic differentiation of PDLSCs and further probe the potential role of miR-23b in this process, in an effort to provide a new potential therapeutic target for periodontitis.

## Materials and methods

### Cell culture

All the cell culture procedures were authorized by the ethics committee of Xi'an Jiaotong University Stomatology Hospital. HPDLSCs were isolated and cultured as previously described 15, 16. In brief, healthy third molars were obtained after obtaining informed consent from female patients aged 18-24. The obtained third molars were washed thoroughly with phosphate buffered saline (PBS) (P5493, Sigma-Aldrich, USA). Then, the periodontal ligament tissue in the middle third of the third molar root was scraped and treated with 3 mg/mL collagenase type I (SCR103, Sigma-Aldrich, Missouri, USA) at 37°C for 1 h. Cell suspensions were incubated at 37°C in 5% CO_2_ using α-MEM (32561102, ThermoFisher, Massachusetts, USA) containing 20% fetal bovine serum (F8687, Sigma-Aldrich, USA). Cells were cultured in standard medium (α-MEM with 10% fetal bovine serum) and passaged at approximately 80%-90% confluence, and the cultured third passage cells were used for subsequent experimental studies.

### Flow cytometry analysis

Flow cytometry was performed according to the manufacturer's instructions (BD Biosciences, San Jose, CA, USA) to detect the immunophenotype of PDLSCs. Briefly, 1 × 10^6^ hPDLSCs were washed 3 times with PBS (Thermo Fisher Scientific, Waltham, MA, USA). Then, cells were resuspended with 500 µL of PBS and aliquoted into six 1.5 mL Eppendorf (EP) tubes. MSC-positive markers (Stro1-PE, CD105-PE, and CD29-PE) and MSC-negative markers (CD34-PE and CD45-PE) antibodies were then added to each tube, respectively. After 30 min incubation in the dark at 37°C, unbound antibody was washed away with PBS and detected by flow cytometry.

### Cell transfection

For miRNA transfection, hPDLSCs were transfected as previously reported 17, 18. MiR-23b-3p mimics, negative control mimics (mimics NC), the miR-23b-3p inhibitor, and negative control inhibitor (inhibitor NC) were synthesized from RiboBio (Guangzhou, China). Briefly, Lipofectamine 2000 (Invitrogen, USA) was mixed with miR-23b-3p in serum-free α-MEM for 20 min according to the manufacturer's instructions and then transfected in complete α-MEM. After 48 h, the transfected cells were used for subsequent experiments. Overexpression and short hairpin RNAs (shRNAs) lentivirus were designed and synthesized by GenePharma (Shanghai, China). The sh-SNHG5 (knockdown SNHG5), overexpression lentivirus (SNHG5), and sh-NC (negative control) were executed to regulate SNHG5 expression in hPDLSCs. The Polyfect-C (Inovogen, China) transfection reagent was used for SNHG5 overexpression. The Runx2 plasmids (Runx2) and empty plasmid (Runx2-EV) were constructed by RiboBio (Guangzhou, China) and executed to regulate Runx2 expression in hPDLSCs. For transfection, SNHG5 lentivirus and Runx2 plasmids were transfected according to manufacturer's instructions, and Puromycin Dihydrochloride (Beyotime Biotechnology, China) was executed to construct stable SNHG5 transfection cells. Then, the transfection efficiency was identified by RT-qPCR and western blot.

### RNA purification and quantitative real‑time PCR

Total RNA from hPDLSCs was extracted using TRIzol reagents (Thermo Fisher Scientific USA) according to the Thermo manufacturer's instructions. First-strand cDNA was synthesized from 500 ng of total RNA using the HiScript Reverse Transcriptase reagent kit (vazyme, China). The amplification conditions were: denaturation at 95 °C for 120 s, 40 cycles of denaturation at 95 °C for 10 s, annealing at 60 °C for 30 s, and final extension at 70 °C for 5 min. qRT-PCR was analyzed using the 2^-∆∆Ct^ method. U6 or GAPDH were used as endogenous normalization controls. The sequences of primers are shown in Table 1.

### Western blot analysis

Cells were washed with pre-chilled PBS and lysed in radioimmunoprecipitation assay (RIPA) buffer. Then, protein samples were separated by 10% SDS-PAGE (Beyotime Biotechnology, China), and the separated proteins were transferred to PVDF membranes (Millipore, Germany) at 110 mV. PVDF membranes were blocked in 5% nonfat milk for 1 hr at room temperature, rinsed with PBS, and incubated with target antibody and GAPDH antibody (1:5,000; Abcam, UK) overnight at 4°C. Afterwards, they were incubated with secondary antibodies (Proteintech, USA) for 2 h. After rinsing the secondary antibody, the PVDF membrane was detected by chemiluminescence and quantified using ImageJ.

### Alizarin red staining and oil red O staining

The osteogenic induction medium consisted of α-MEM, 10 mM β-glycerophosphate, 0.1 μM dexamethasone, and 50 μg/mL ascorbic acid (Sigma, USA). Adipogenic induction medium consisted of α-MEM, 0.5 mM IBMX, 200 μM indomethacin, 1 μM dexamethasone, and 10 μg/mL insulin (Sigma, USA). After induction of osteogenic differentiation of hPDLSCs in osteogenic differentiation medium for 3 weeks, cells were fixed with 4% paraformaldehyde for 30 min, then rinsed with PBS, and then stained with 0.1% alizarin red (ARS; pH 4.2). After rinsing them again with PBS, they were dissolved in 10% cetylpyridinium chloride and analyzed at 562 nm absorbance using a spectrophotometer to quantify ARS. hPDLSCs were induced to differentiate in adipogenic differentiation medium for 4 weeks. Cells were fixed with 4% paraformaldehyde for 30 minutes, then washed with PBS, stained with 0.1% Oil Red O for 30 minutes, and then observed under the microscope for intracellular lipid droplet formation.

### Dual luciferase reporter assays

The binding sites between SNHG5 or Runx2 and miR-23b-3p were predicted using starBase and targetscan databases. The luciferase plasmids containing wild-type SNHG5 (WT-SNHG5), mutant of SNHG5 (MUT-SNHG5), Runx2 3′ untranslated region (UTR)-WT, or Runx2 3′UTR-MUT were was synthesized, cloned into the downstream of the luciferase coding sequence in pmirGLO plasmids (Promega, Madison, USA). hPDLSCs were co-transfected with miR-23b-3p mimic or a vector, together with *Renilla* plasmid (Promega, Madison, USA), and above luciferase plasmids reporter with Lipofectamine 2000 reagent (Invitrogen, USA). The luciferase activity was detected with the dual luciferase reporter assay system (Promega, Madison, USA) after 48 h of transfection. The firefly luciferase activity was normalized to *Renilla*.

### Fluorescence *in situ* hybridization (FISH)

FISH assays were performed using a FISH kit (Ribo Bio) according to the manufacturer's instructions as previously described 19. Briefly, cells were fixed in 4% paraformaldehyde for 15 min at room temperature and washed with PBS. Then, cells were permeabilized in 0.5% Triton X-100 for 10 min and washed extensively with PBS. Next, cells were prehybridized at 55 °C for 40 min. For hybridization, the anti-SNHG5 probe was mixed with the hybridization solution and added to the cells overnight at 37°C in the dark. Then, cells were washed 3 times with PBS, nuclei were counterstained with DAPI (Beyotime Biotechnology, China), and images were acquired using a fluorescence microscope (Olympus, Japan).

### RNA pull-down assay

The RNA-binding protein-immunoprecipitation kit (Millipore, USA) was used for RIP analysis. In short, the wide type (Bio-miR-23b-3p-WT) or mutant (Bio-miR-23b-3p-Mut) sequences of miR-23b-3p containing the potential binding sites of SNHG5 were synthesized and labeled by biotin. Bio-NC was seen as the negative control. Cells were lysed in RIPA buffer (Beyotime, Guangzhou, China) and the supernatant was treated with Bio-miR-23b-3p-WT, Bio-miR-23b-3p-Mut or Bio-NC at 4 °C for 2 h. Thereafter, 50 μl of M-280 streptavidin magnetic beads (Invitrogen, USA) was mixed with the incubation for 1 h. As for Biotin pull-down assays, SNHG5 and its antisense RNA were biotin-labeled with the Biotin RNA Labeling Mix (Roche Diagnostics, Indianapolis, IN) and purified with a RNeasy Mini Kit (Qiagen, Valencia, CA). The coprecipitated RNAs were extracted and quantified by qRT-PCR analysis.

### Statistical Analysis

Statistically significant differences were determined using two-tailed Student's t-test or ANOVA. Data represented the mean ± SD of at least n = 3 independent experiments. *p < 0.05.

## Results

### Expression of SNHG5 and miR-23b-3p during osteogenic differentiation of hPDLSCs

**Figure 1:**
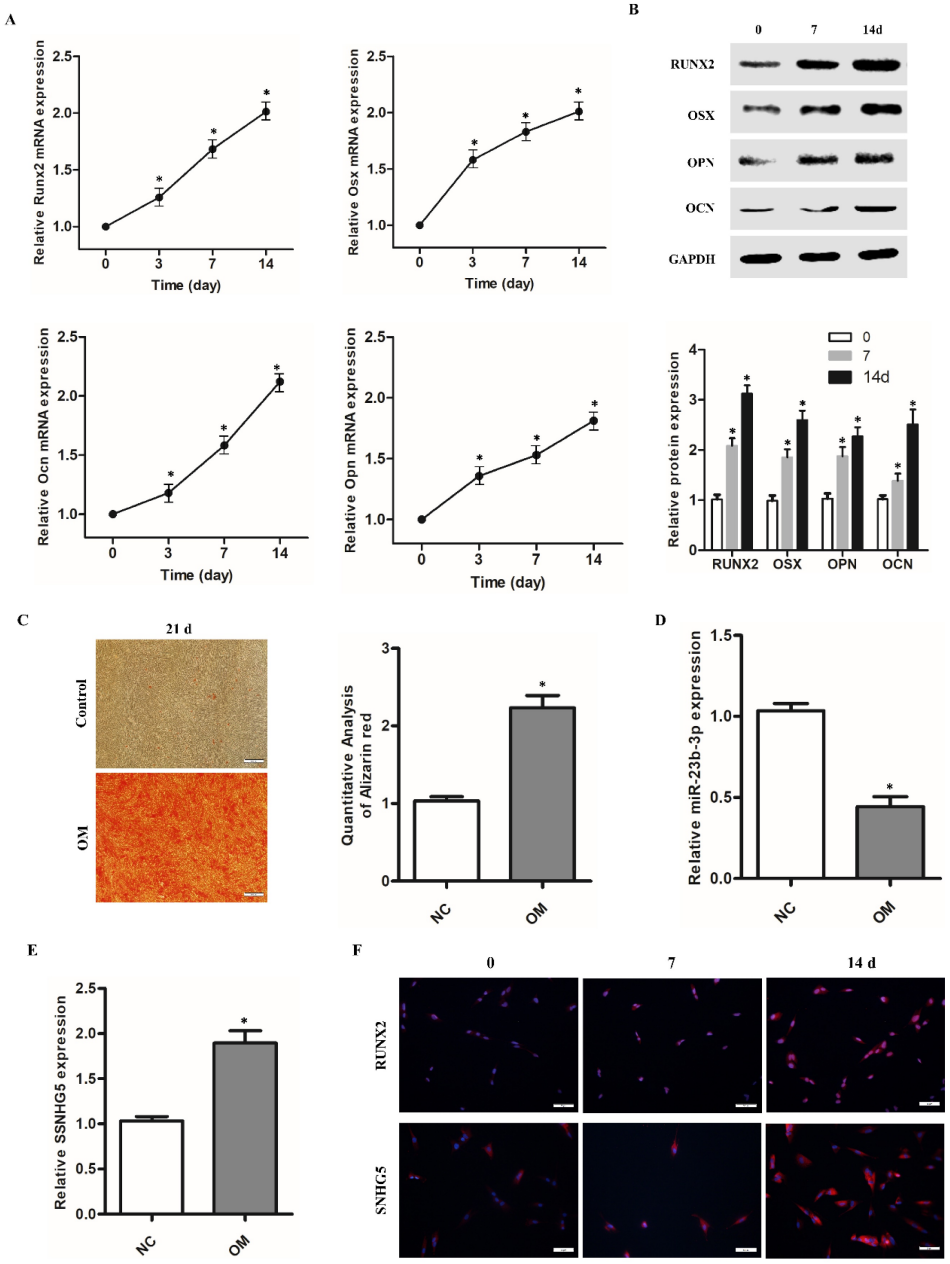
SNHG5 and miR-23b-3p expression in hPDLSCs. (A) *Runx2*,* Osx*, *Ocn*, and *Opn* expression after stimulation with osteogenic medium (OM) for 2weeks in hPDLSCs. (B) RUNX2, Osx, OCN, and OPN protein expression was detected by Western blot after stimulation with OM for 7 days and14 days in hPDLSCs. (C) Alizarin red staining and its quantification results after stimulation with OM for 21 days. (D) miR-23b-3p and (E) SNHG5 expression was detected by qRT-PCR. (F) Expression of SNHG5 and RUNX2 after stimulation with OM for 7 days and14 days in hPDLSCs were detected by Fluorescence *in situ* hybridization (FISH). *p < 0.05.

To elucidate the expression of SNHG5 and miR-23b-3p, hPDLSCs were harvested at different time points during the 14-day osteogenic induction. qRT-PCR analysis revealed that the expression of OCN, OSX, and Runx2 mRNA gradually increased due to prolonged osteogenic induction (Figure 1A). Western blot analysis confirmed their significant upregulation at the protein level (Figure 1B). Likewise, Alizarin Red S staining assay showed that mineralization was significantly enhanced with the induction of hPDLSC osteogenic differentiation (Figure 1C). Most importantly, we observed that the levels of SNHG5 and RUNX2 increased with the time of osteogenic induction (Figure 1A, E). The trend for SNHG5 and RUNX2 was further confirmed by fluorescence *in situ* hybridization (FISH; Figure 1F). However, qRT-PCR analysis showed that the expression of miR-23b-3p was gradually and significantly downregulated with the induction of osteogenic differentiation of hPDLSCs. These data suggest that there is a negative linear relationship between SNHG5 and miR-23-3p, that is, the expression of SNHG5 and miR-23-3p is negatively correlated during hPDLSC osteogenic differentiation (Figure 3A). Overall, these findings suggest that SNHG5 and miR-23b-3p may be involved in regulating the osteogenic differentiation of hPDLSCs.

### Effects of SNHG5 on osteogenic differentiation of hPDLSCs

**Figure 2:**
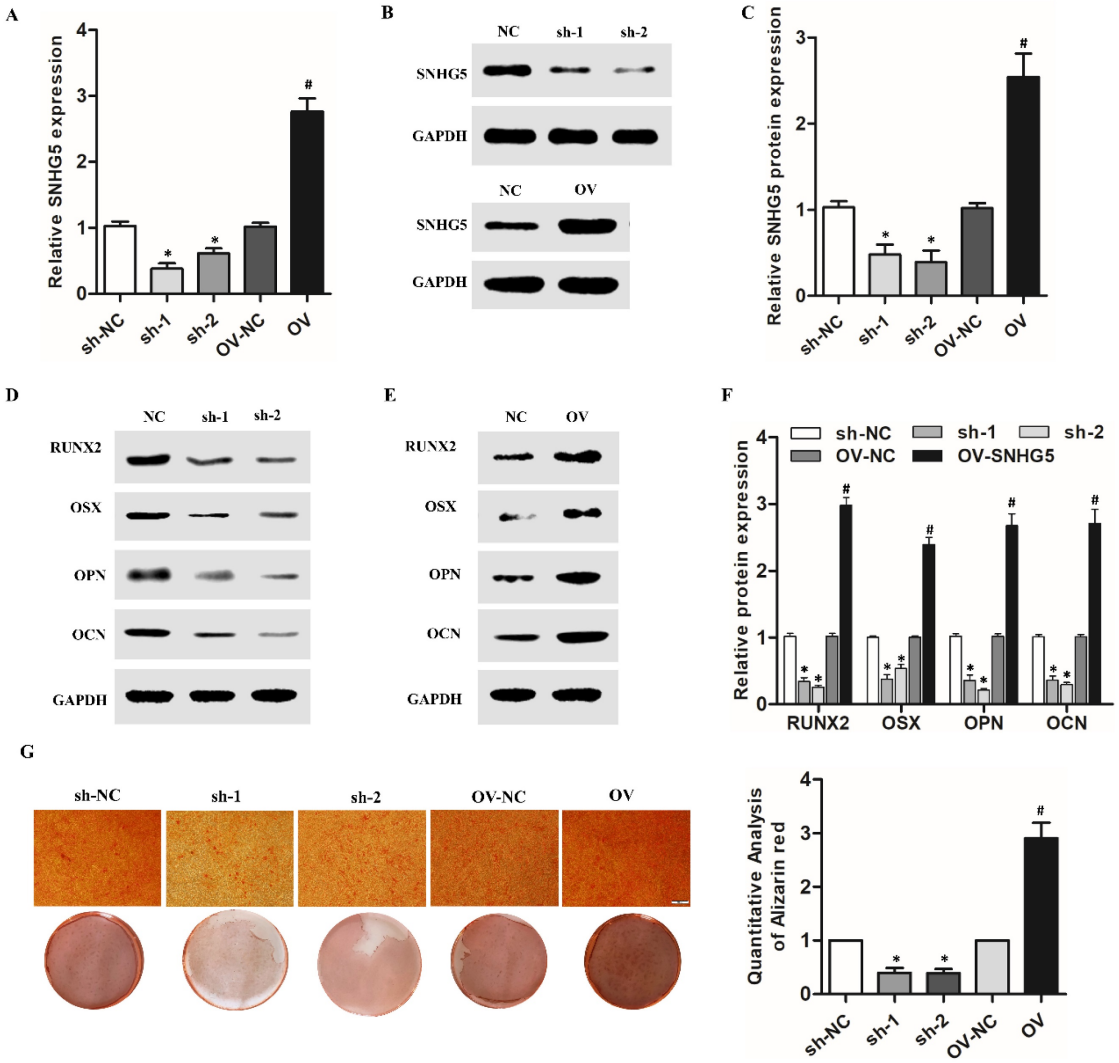
Effects of SNHG5 on osteogenic differentiation of hPDLSCs. (A, B, C) SNHG5 expression was assessed 72 h after shRNA and overexpression (OV) lentivirus transfection by qRT-PCR (A) and Western blot (B, C). (D) Expression of osteogenesis-related proteins (i.e., RUNX2, OSX, OCN, and OPN) after SNHG5 shRNA transfection in hPDLSCs. (E) Expression of osteogenesis-related proteins after SNHG5 overexpression in hPDLSCs. (F) The quantification of osteogenesis-related proteins expression in above groups. (G) Alizarin Red S staining was performed on hPDLSCs to investigate the effects of SNHG5 on the osteogenic differentiation of hPDLSCs. Scale bar = 100 μm. sh-NC: SNHG5 shRNA negative control; sh-1 and sh-2: SNHG5 shRNA; OV-NC: SNHG5 overexpression negative control; OV: SNHG5 overexpression. * *p* < 0.05 compared with the sh-NC group; ^#^*p* < 0.05 compared with the OV-NC group.

SNHG5 overexpression and shRNA lentivirus were used to regulate the expression of SNHG5 in hPDLSCs to investigate its role in the osteogenic differentiation of hPDLSCs, and its expression was identified by qRT-RCP and western blotting. As shown in Figure 2A-C, with sh-SNHG5 or OV-SNHG5 transfection, the level of SNHG5 was significantly decreased or increased compared with the control group (p < 0.05), thus indicating that the transfection was successful. Western blotting showed that overexpression of SNHG5 in hPDLSCs resulted in the upregulation of RUNX2, OCN, OSX, and OPN, but SNHG5 knockdown inhibited their expression (Figure 2 D-F). ARS assay (Figure 2G) results further indicated that SNHG5 overexpression promoted osteogenic differentiation of hPDLSCs, and vice versa.

### MiR-23b-3p was directly sponged by SNHG5 in hPDLSCs

**Figure 3:**
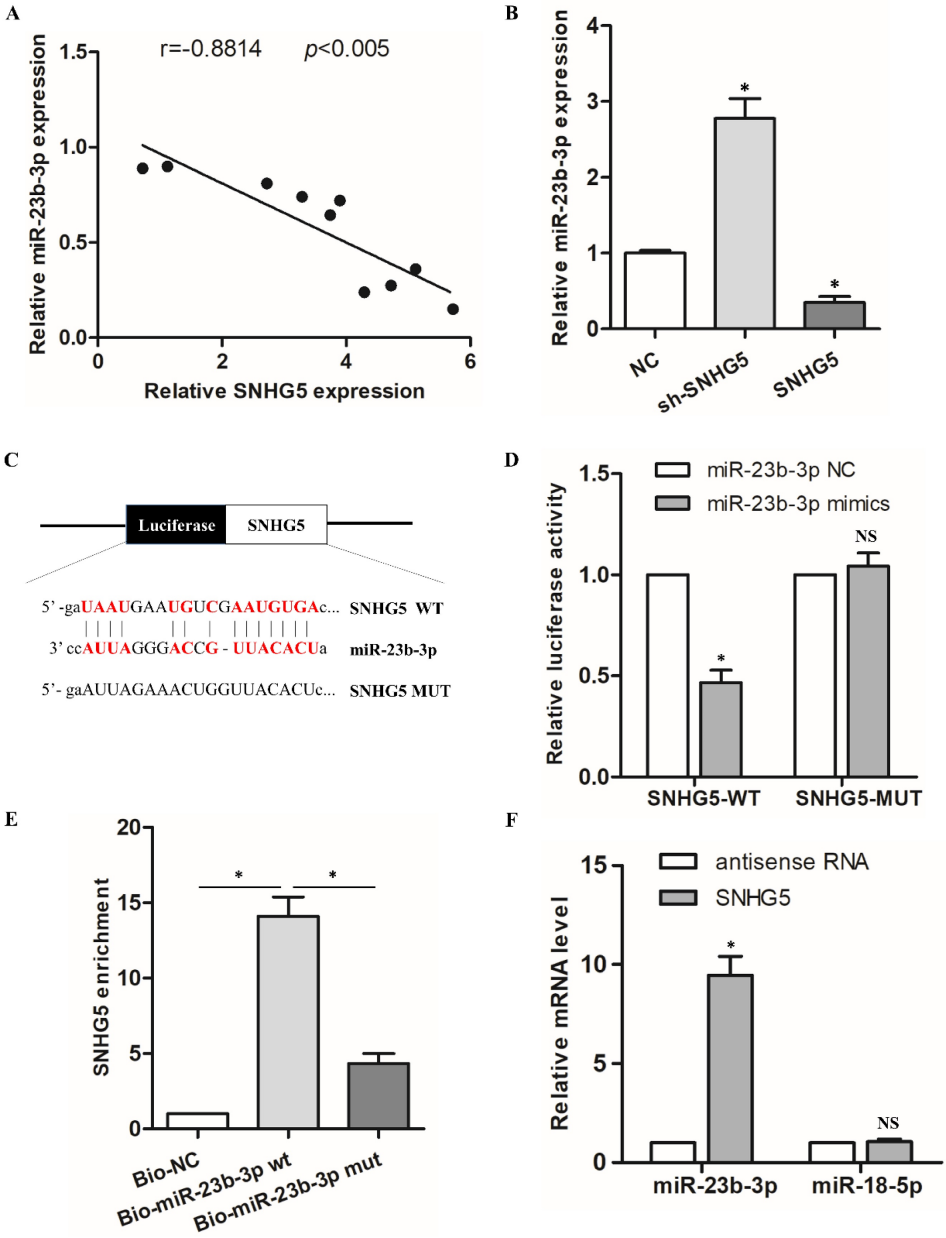
MiR-23b-3p was directly sponged by SNHG5 in hPDLSCs. (A) Relationship between SNHG5 and miR-23b-3p in hPDLSCs. (B) miR-23b-3p expression was detected by qRT-PCR in sh-SNHG5, NC, and SNHG5-overexpressing (SNHG5) hPDLSCs. (C) Luciferase reporter assay results of SNHG5 and its mutant (SNHG5-MUT). (D) Luciferase activity results of SNHG5-WT and SNHG5-MUT in miR-23b-3p mimics or the NC group. (E) RNA pull-down assay elucidated that SNHG5 could be enriched by biotin-labelled miR-23b-3p but not biotin-labelled miR-23b-3p mutant type. (F) Lysates from hPDLSCs were incubated with biotin-labeled SNHG5 and antisense RNA for biotin pull-down assay to examine miR-23b-3p and miR-218-5p levels by qRT-PCR. MiR-218-5p was set as a negative control. *p < 0.05, NS: no statistical significance

To elucidate the elaborate mechanism of SNHG5 in hPDLSCs osteogenic differentiation, we predicted the potential targets of SNHG5. Interestingly, there was a remarkable inverse correlation between SNHG5 and miR-23b-3p expression during the osteogenic differentiation of hPDLSCs (r = -0.6834,* p* < 0.005; Figure 3A). qRT-PCR results demonstrated that miR-23b-3p is dramatically reduced in SNHG5-overexpressing hPDLSCs but upregulated in sh-SNHG5 hPDLSCs (Figure 3B). To identify whether SNHG5 directly modify miR-23b-3p, we constructed SNHG5 (SNHG5-wt) and mutated (SNHG5-mut) luciferase reporters (Figure 3C). The results revealed that overexpression of miR-23b-3p obviously suppresses SNHG5-WT luciferase activity. No manifest impact was detected in SNHG5-MUT (Figure 3D), thus indicating a direct interaction between SNHG5 and miR-23b-3p in a sequence-specific manner. In addition, the biotinylated SNHG5 probe and SNHG5 biotinylated antisense DNA probe were synthesized to pull down miR-23b-3p. MiR-218-5p was set as a negative control, due to it was formed no base pairing with SNHG5. The results elucidated that SNHG5 specifically pulled down miR-23b-3p (Figure 3E, F), however, SNHG5 was not able to pull down miR-218-5p (Figure 3F). These data confirmed that SNHG5 physically interacts with miR-23b-3p at the putative binding site. In summary, our results supply convincing evidence that SNHG5 negatively regulates miR-23b-3p expression by directly binding to its site.

### MiR-23b-3p inhibited hPDLSCs osteogenic differentiation by targeting *Runx2*

**Figure 4:**
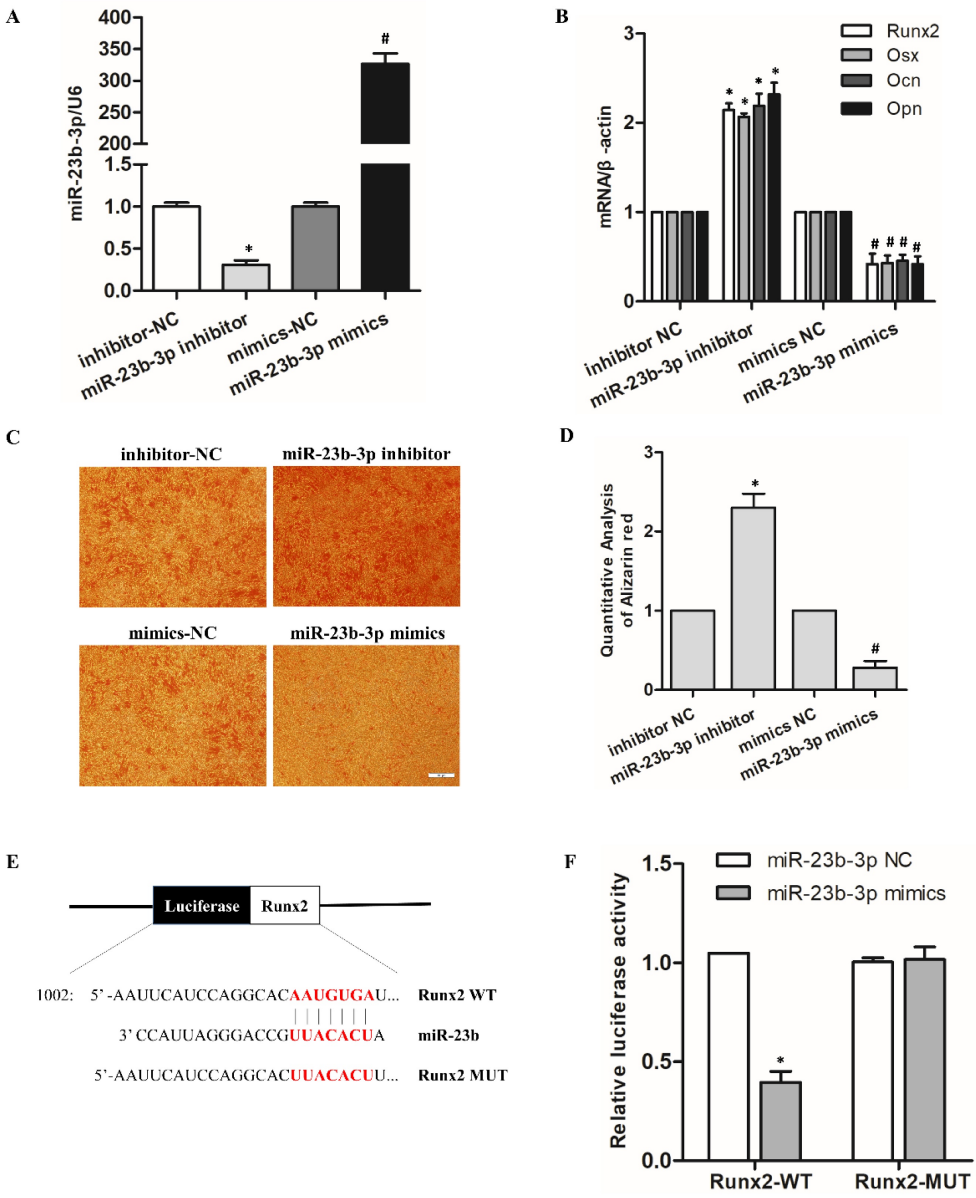
MiR-23b-3p inhibited hPDLSC osteogenic differentiation by targeting *Runx2*. (A) qRT-PCR analysis indicated miR-23b-3p expression in hPDLSCs after transfection with miR-23b-3p mimics or the miR-23b-3p inhibitor for 48 h. (B) Expression of the osteogenesis-related genes *Runx2*,* OSX*, *Ocn*, and *Opn* in in the indicated groups after stimulation with OM for 7 days. (C, D) Alizarin Red S staining was performed on hPDLSCs to investigate the effects of miR-23b-3p on the osteogenic differentiation of hPDLSCs. Scale bar = 100 μm. (E) Schematic representation of luciferase reporter plasmid containing wild-type *Runx2* (*Runx2*-WT) and a mutant reporter (*Runx2*-MUT), and mutated bases and potential binding site are indicated in red. (F) Luciferase activity of *Runx2*-WT and *Runx2*-MUT in miR-23b-3p mimics or the NC group. * *p* < 0.05 compared with the inhibitor control group; ^#^*p* < 0.05 compared with the mimic control group.

To illustrate the function and mechanism of miR-23b-3p in hPDLSCs. MiR-23b-3p inhibition (inhibitor) and miR-23b-3p overexpression (mimic) vectors were constructed and transfected into hPDLSCs. The results investigated that miR-23b-3p obviously increases in mimic-transfected cells and vice versa (Figure 4A). qRT-PCR results demonstrated that miR-23b-3p inhibitor promoted the expression of the osteoblastic markers *Runx2*,* Osx*, OCN and *OPN*, while miR-23b-3p mimics reversed OM-induced osteogenic differentiation (Figure 4B). ARS assays also verified that miR-23b-3p inhibitor induces whereas miR-23b-3p mimics inhibit the osteogenic differentiation of hPDLSCs (Figures 4C, 4D). qRT-PCR results manifested that *Runx2* expression noticeably decreases in miR-23b-3p mimics but increases in the miR-23b-3p inhibitor (Figure 4B). Figure 4E indicated that the *Runx2* 3′-UTR has a potential binding site for miR-23b-3p. The luciferase reporter gene assay illustrated that the miR-23b-3p mimics had no effect on the *Runx2*-mutant form, but dramatically inhibited the luciferase activity of *Runx2*-WT (Figure 4F). Overall, these data confirm that miR-23b-3p suppresses the expression of *Runx2* via directly binding to the *Runx2* 3′-UTR.

### SNHG5 modulated Runx2 expression and osteogenic differentiation of hPDLSCs through functioning as a sponge for miR‐23‐3p

**Figure 5:**
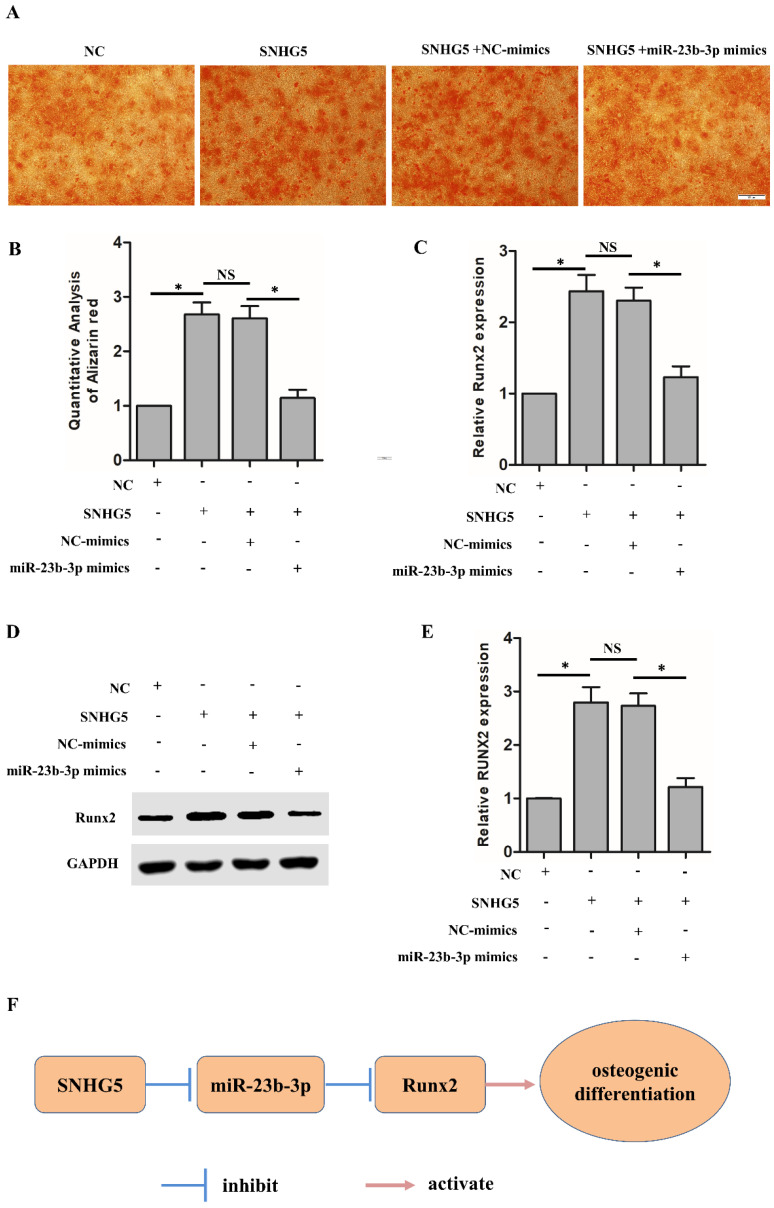
SNHG5 regulates Runx2 expression and osteogenesis in hPDLSCs by suppressing miR-23b-3p. (A, B) Alizarin red staining results in the indicated groups after stimulation with OM for 21 days. Scale bar = 100 μm. (C) *Runx2* mRNA expression was measured by qRT-PCR in the indicated groups. (D, E) *Runx2* protein expression was analyzed by Western blot in the indicated groups. (F) Schematic overview of the function and mechanism of SNHG5 in hPDLSC osteogenesis. *p < 0.05

Next, we further explored whether SNHG5 regulates *Runx2* expression through functioning as a sponge for miR-23b-3p. As shown in Figure 5A, SNHG5 overexpression dramatically increased the osteogenic differentiation of hPDLSCs, and co-transfection of miR-23b-3p mimics reversed the observed SNHG5-induced hPDLSCs osteogenic differentiation. qRT-PCR analysis illustrated that SNHG5 dramatically upregulates* Runx2* mRNA expression and that co-transfection of miR-23b-3p mimics reverses* Runx2* expression (Figure 5C). In agreement with the mentioned findings, the Western blot confirmed that the RUNX2 protein level change (Figures 5D, 5E). In short, our study reveals that SNHG5 inhibits miR-23b-3p expression to promote hPDLSCs osteogenesis differentiation by promoting the expression of *Runx2* (Figure 5F).

## Discussion

Periodontitis, which destroys the bone structure that supports teeth, has become a major public health burden 20. However, the treatment of periodontitis requires plaque control and intensive lifestyle interventions, such as smoking cessation 21. Although these treatments are feasible in some cases, new and effective treatments are urgently needed. PDLSCs are hopeful tissue engineering seed cells for periodontal tissue regeneration 22. Recent studies have shown that regulating the quantity, distribution and osteogenic differentiation of PDLSC can significantly induce periodontal bone tissue regeneration 23. Therefore, elucidate the mechanism of regulating PDLSCs osteogenic differentiation is one of the important strategies for the treatment of periodontitis. Previous studies on the pathogenesis of periodontitis show that the progression of periodontitis involves a variety of genetic factors 24. The regulatory function of key genes may facilitate the progress of novel targeted therapies. LncRNAs regulate the expression of multiple target genes and play a crucial role in the occurrence and development of a variety of diseases 25. Hence, further exploration of the mechanism of lncRNAs in the PDLSC osteogenic differentiation is necessary to develop novel periodontitis treatment strategies.

Previous studies demonstrated that SNHG5 is involved in the occurrence and pathophysiology of a variety of diseases, especially in bone metabolic diseases 19, 26. Unfortunately, the specific regulatory function and biological mechanism of SNHG5 in PDLSCs remain unclear. In the current study, we determined that the expression of SNHG5 was dramatically upregulated after the osteogenic differentiation of hPDLSCs. Ectopic expression of SNHG5 experiment further clarified that SNHG5 overexpression dramatically promotes hPDLSC osteogenic differentiation, while knockdown of SNHG5 significantly inhibited the osteogenic effect, thereby confirming the stimulative role of SNHG5 in the osteogenic differentiation of hPDLSCs. It is noteworthy that SNHG5 adsorbed miR-212-3p and miR-582-5p in bone marrow mesenchymal stem cells (BMSCs), thereby enhancing their osteogenic effects 7, 19. In view of our current research results on the positive correlation between SNHG5 and miR-23b-3p, we suppose that SNHG5 regulates osteogenic function and mechanism in hPDLSC via sponging miRNA.

MiRNAs, similar to LncRNAs, are small non-coding RNAs involved in a variety of biological processes 27-29. Our previous studies have revealed that miR-23b inhibits osteogenic differentiation of hPDLSCs in TNF‐ α-induced inflammatory microenvironments by adsorbing Runx2 and reducing β-catenin activity 14. In addition, after reviewing the literature and databases, no other related osteogenic genes (OSX/OCN/OPN) were found to have base complementary pairing with miR-23b. Nevertheless, the role of miR-23b in the regulation of osteogenic differentiation of hPDLSCs by SNHG5 and its potential molecular mechanism deserve to be ulteriorly investigated. In the present study, we found that miR-23b is highly expressed in the hPDLSCs knockdown SNHG5, and vice versa. The RNA pull-down and dual luciferase report assay revealed the cause of the negative correlation between miR-23b and SNHG5—base complementarity and sponge adsorption between miR-23b-3p and SNHG5. Our further results confirmed that miR-23b-3p inhibits osteogenic differentiation of stem cells by sponge adsorption of Runx2. We further investigate whether SNHG5 regulates Runx2 expression and hPDLSC osteogenic differentiation through sponging miR-23b-3p. The results clarified that SNHG5 overexpression dramatically increases the expression of Runx2 and that co-transfection with miR-23b-3p mimics reverses the promotive effect. Besides, miR-23b-3p also reversed the osteogenesis effect of hPDLSCs induced by SNHG5 overexpression. These results elucidated that SNHG5 regulates Runx2 expression via sponging miR-23b-3p, thereby inducing the osteogenic differentiation of hPDLSCs. It is worth noting that, SNHG5 promotes the osteogenic differentiation of BMSCs via the miR-212-3p/GDF5/SMAD pathway or miR-582-5p/RUNX3 axis 7, 19. These results suggest that SNHG5 may play a powerful role in promoting osteogenic differentiation through multiple pathways.

In conclusion, our study elucidated that SNHG5 executed the promoting role in the osteogenic differentiation of hPDLSCs. Mechanistically, SNHG5 increases Runx2 expression in hPDLSCs by competitively binding to miR-23b-3p, thus promoting osteogenic differentiation of hPDLSCs. Our study reveals the specific molecular mechanism of SNHG5 in hPDLSC osteogenic differentiation, suggesting that SNHG5 could be a potential target for periodontitis therapy.

## Supplementary Material

Supplementary figure.

## Figures and Tables

**Table 1:** Nucleotide sequence of primers used in qRT-PCR.

Gene	Forward primer sequences 5′-3′	Reverse primer sequences 5′-3′
Runx2	CCGCCTCAGTGATTTAGGGC	GGGTCTGTAATCTGACTCTGTCC
Ocn	CACTCCTCGCCCTATTGGC	CCCTCCTGCTTGGACACAAAG
Opn	GGAGTTGAATGGTGCATACAAGG	CCACGGCTGTCCCAATCAG
Osx	CAGGCTATGCTAATGATTACC	GGCAGACAGTCAGAAGAG
SNHG5	GTGGACGAGTAGCCAGTGAAG	GCCTCTATCAATGGGCAGAC
miR-23b-3p	CGCATCACATTGCCAGGG	GTGCAGGGTCCGAGGT
U6	CGGCGGTCGTGAAGCGTTCCAT	CCAGTGCAGGGTCCGAGGTAT
GAPDH	CCTGCACCACCAACTGCTTA	GGCCATCCACAGTCTTCTGAG
