# Supplementary material for: Long non‑coding RNA SNHG5 promotes osteogenic differentiation of human periodontal ligament stem cells via mediating miR‑23b‑3p/Runx2 axis
Source: Int J Med Sci. 2023 May 21;20(7):958–68. doi: 10.7150/ijms.82454 (PMC10266046; doi:10.7150/ijms.82454)
Supplement: Supplementary file 1 — Supplementary figure. [file ijmsv20p0958s1.pdf]

Supplemental figure 1

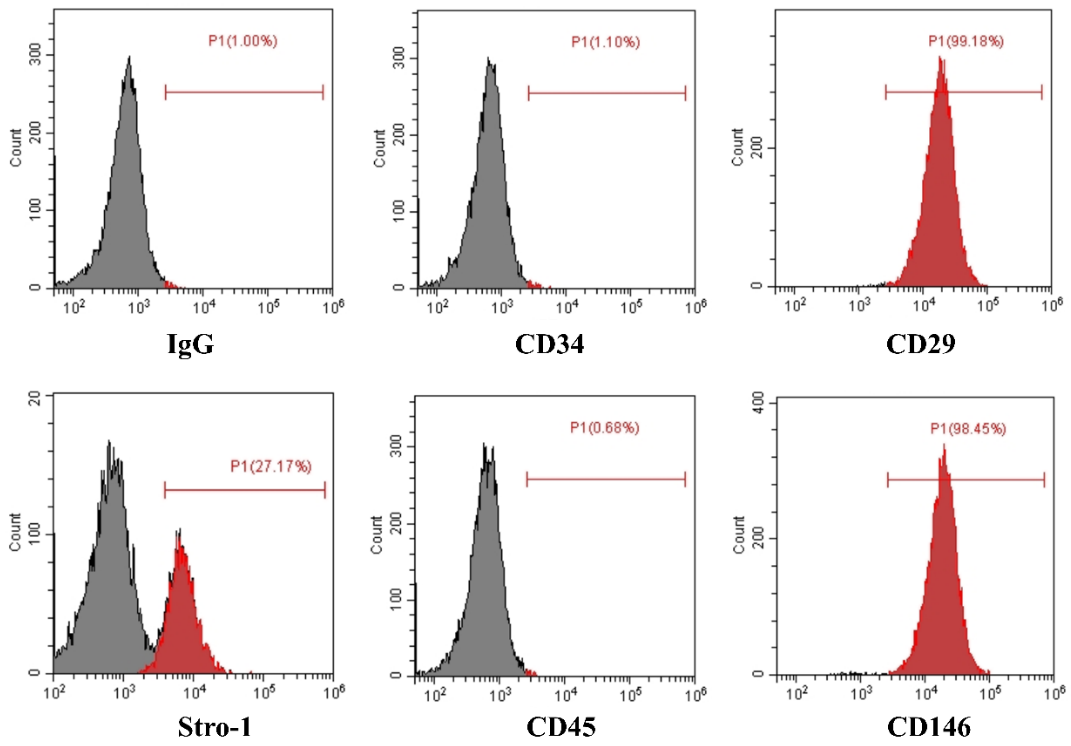

Supplemental figure 1. The Flow cytometry analysis of PDLSCs. Cell marker expression was measured through flow cytometry. CD29, CD146 and Stro-1 were positively expressed in PDLSCs, while CD34 and CD45 were negatively expressed in PDLSCs. IgG was used as a negative control.
